# The e13a3 (b2a3) and e14a3 (b3a3) BCR::ABL1 isoforms are resistant to asciminib

**DOI:** 10.1038/s41375-024-02314-7

**Published:** 2024-06-15

**Authors:** Inga B. Leske, Oliver Hantschel

**Affiliations:** Institute of Physiological Chemistry, Faculty of Medicine, Philipps University of Marburg, 35032 Marburg, Germany

**Keywords:** Chronic myeloid leukaemia, Preclinical research, Oncogenesis, Chronic myeloid leukaemia, Cancer therapeutic resistance

## To the Editor:

**Fig. 1:**
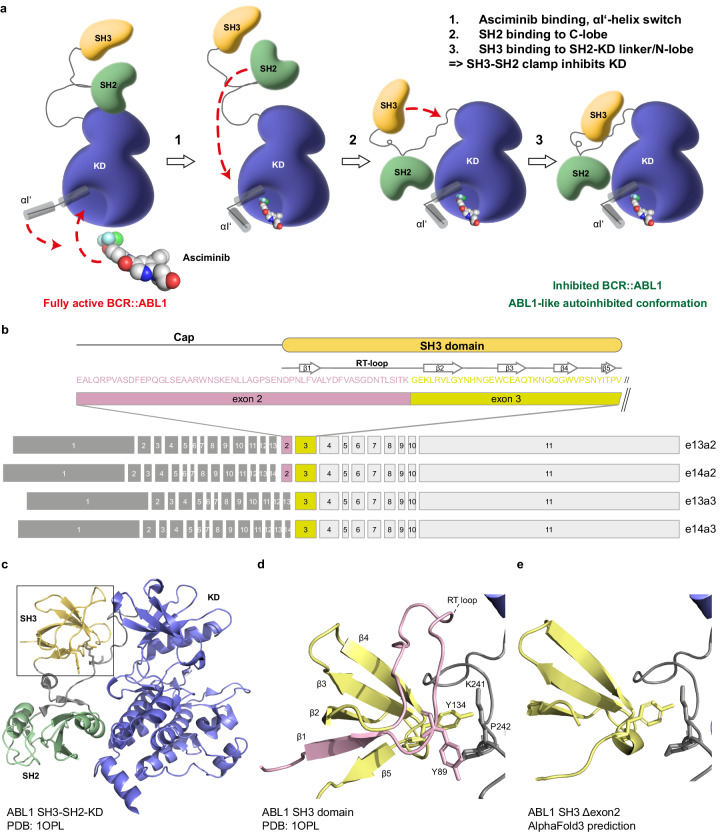
Mechanism-of-action of asciminib and structural consequences of lack of ABL1 exon 2 in BCR::ABL1 e13a3 and e14a3 on the SH3 domain structure and function. **a** Schematic representation of the mechanism-of-action of BCR::ABL1 inhibition by asciminib. Only the three core domains in ABL1 that are necessary for asciminib-mediated inhibition, SH3 domain (yellow), SH2 domain (green) and kinase domain (KD; blue), are shown. BCR and other ABL1 domains are not shown for graphical convenience. Binding of asciminib to the fully activated BCR::ABL1 kinase results in a conformational change in the αI’ helix (step 1), which enables engagement of the SH2 domain with the C-lobe of the KD (step 2). This results in interaction between the SH3 domain, SH2-KD-linker and KD N-lobe (step 3), which ultimately leads to inhibition of BCR::ABL1 kinase activity through the SH3-SH2-clamp. **b** Overview of BCR::ABL1 exon organization of the common e13a2, e14a2 transcript variants, as well as e13a3 and e14a3 that lack ABL1 exon 2. The amino acid sequence of ABL1 exon 2 (pink) and exon 3 (lemon) is shown, with the secondary structure elements of the ABL1 SH3 domain displayed above. **c** Cartoon representation of the crystal structure of the human ABL1 in its autoinhibited conformation (PDB entry 1OPL, molecule A) highlighting the close interface of the SH3 domain with the KD N-lobe sandwiching the SH2-KD linker. Domains are colored as in panel **a**. **d** Close-up view on the structure of the ABL1 SH3 domain. Amino acids encoded by exon 2 and exon 3 are colored in pink and lemon, respectively, as in panel **b**. Residues Tyr-89 and Tyr-134 are important for the interaction with Lys-241 and Pro-242 in the SH2 KD linker (grey) and shown as sticks. The β1-strand and the RT loop, which are important for ligand recognition and missing in BCR::ABL1 e13a3 and e14a3, are shown in pink. **e** Superimposition of the AlphaFold3 model of the ABL1 SH3 domain missing the exon 2 encoded region on the crystal structure of ABL1. The crystal structure of the full-length SH3 domain is hidden.

Asciminib is a first-in-class allosteric BCR::ABL1 inhibitor and was recently approved for the treatment of adults with Philadelphia (Ph) chromosome-positive chronic myeloid leukaemia (CML) in chronic phase, who have previously been treated with two or more tyrosine kinase inhibitors (TKIs) [1]. In addition, the superior efficacy and excellent safety and tolerability of asciminib in newly diagnosed CML patients when compared to conventional (ATP-competitive) TKIs is expected to extend asciminib approval to first line treatment [2]. Asciminib is a highly selective and potent inhibitor of kinase activity and signalling of BCR::ABL1 and acts by binding to the allosteric myristoyl binding pocket of the ABL1 tyrosine kinase domain [3]. The major splice isoform 1b of ABL1 is N-terminally myristoylated. Intramolecular binding of the myristoyl moiety to its hydrophobic binding pocket in the kinase domain is required for docking of the SH3-SH2 domain clamp to the kinase domain. This interaction ensures that the proto-oncogene ABL1 is autoinhibited most of the time in cells [4, 5]. As the Ph chromosome translocation replaces the first exon of ABL1, including the myristoylation site, with BCR sequences, the myristoyl binding pocket in the kinase domain lacks its endogenous ligand. This contributes to kinase activation, constitutive signaling and oncogenicity of BCR::ABL1 [6]. Asciminibs’ unique mechanism of inhibition requires binding to the myristoyl pocket, setting off a conformational switch of the most C-terminal α-helix of the kinase domain (α-I’-helix), which enables binding of the SH3-SH2 domain clamp to the kinase domain resulting in BCR::ABL1 inhibition (Fig. 1a). Hence, the functional integrity of both the SH3 and SH2 domains are absolutely required for BCR::ABL1 inhibition by asciminib [7].

Different chromosomal breakpoints within the BCR and ABL1 genes result in the expression of BCR::ABL1 transcript variants and consequently protein isoforms [8]. e13a2 (b2a2) and e14a2 (b3a2) are the most common BCR::ABL1 isoforms in CML patients and contain the complete sequence of the ABL1 SH3, SH2 and kinase domains (Fig. 1b). In contrast, BCR::ABL1 isoforms that lack ABL1 exon 2, most commonly e13a3 (b2a3) and e14a3 (b3a3), are found in <1% of CML cases (Fig. 1c) [9]. While emerging evidence showed asciminib resistance conferred by point mutations in the SH3-SH2-kinase domain unit of BCR::ABL1, it is unknown whether all BCR::ABL1 isoforms are equally sensitive to asciminib [10, 11]. ABL1 exon 2 encodes for 35 amino acids of the unstructured Cap-region, but also for 23 amino acids of the SH3 domain (Fig. 1b). As the SH3 domain is needed for asciminib-mediated inhibition of BCR::ABL1 (Fig. 1a, c) and SH3 point mutations, such as Y115N and P223S, confer asciminib resistance, the lack of ABL1 exon 2 in e13a3 and e14a3 may result in loss of a functional SH3 domain, which we predict cannot be inhibited by asciminib. The possible lack of asciminib sensitivity of e13a3 and e14a3 would therefore constitute a case of primary drug resistance. In contrast, we expect that e13a3 and e14a3 would have normal sensitivity to ATP-competitive TKIs, in line with a previous report [12].

To test this hypothesis, we first studied the impact of the lack of exon 2 on the SH3 domain structure. Sequence analysis and AlphaFold modelling showed that the first β-strand and the entire RT loop are missing (Fig. 1b, d). Both elements are critical for SH3 ligand recognition and ABL1 autoinhibition (Fig. 1c–e) [13]. In addition, it is also unlikely that an SH3 domain lacking the exon 2-encoded sequences would fold. Therefore, lack of exon 2 precludes function and likely native folding of the ABL1 SH3 domain.

**Fig. 2:**
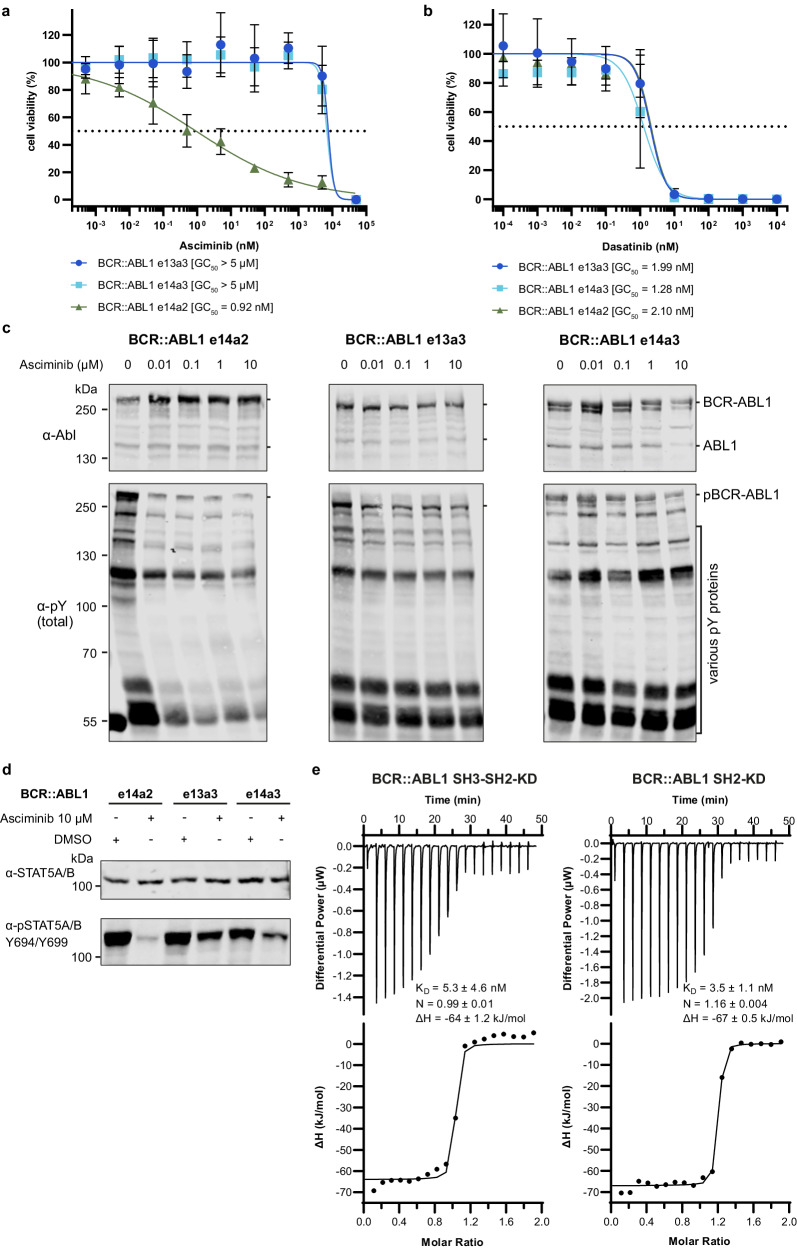
BCR::ABL1 e14a2 and e14a3 are asciminib resistant, but sensitive to dasatinib. Normalized viability of Ba/F3 cells expressing BCR::ABL1 e14a2, e14a3 and e13a3 fusion transcripts was measured in the presence of the indicated concentrations of asciminib (**a**) or dasatinib (**b**) after 48 h. Each data point in the graphs represents mean ± SD from three independent experiments (*n* = 3) performed in triplicates. The continuous line represents the best fit of the data based on a nonlinear regression with variable slope computed from the Prism software. GC_50_ values were calculated from the fit and are shown below the graphs. **c** Immunoblot analysis of BCR::ABL1 expression levels and total tyrosine phosphorylation levels of Ba/F3 cells expressing BCR::ABL1 e14a2, e14a3 and e13a3 fusion transcripts after treatment with the indicated asciminib concentrations for 4 h. A representative immunoblot from three independent repeats is shown. Quantification of total pY signal normalized with BCR::ABL1 expression is shown in SI (Supplementary Fig. S1). **d** Immunoblot analysis of STAT5A/B phosphorylation and total STAT5A/B protein levels in Ba/F3 cells expressing BCR::ABL1 e14a2, e14a3 and e13a3 fusion transcripts upon treatment with either asciminib 10 µM or DMSO for 4 h. A representative immunoblot from three independent repeats is shown. Quantification of pSTAT5 signal normalized with total STAT5 expression is shown in SI (Supplementary Fig. S2). **e** Isothermal titration calorimetry (ITC) measurements of recombinant BCR::ABL1 SH3-SH2-kinase domain unit (SH3-SH2-KD) (left panel) and recombinant BCR::ABL1 SH2-kinase domain unit (SH2-KD) (right panel) (10 μM, respectively) with asciminib (100 μM) at 25 °C. Each panel shows the raw heat signal of an ITC experiment (top) and the integrated calorimetric data of the area of each peak (bottom). The continuous line represents the best fit of the data based on a 1:1 binding model computed from the MicroCal software. A representative measurement of two independent experiments is shown with *K*_D_ value, stoichiometry (*N*) and enthalpy (∆*H*) calculated from the fit.

We next wanted to test the asciminib sensitivity of BCR::ABL1 e13a3 and e14a3. Therefore, we generated e13a3 and e14a3 cDNAs and monitored the effects of asciminib on BCR::ABL1 dependent cell proliferation and survival using retrovirally transduced BaF3 cells. In line with a previous report, both isoforms result in transformation to IL3-independent growth, albeit with lower proficiency than e14a2 (ref. [12]. and data not shown). Once IL3-independent growth was established, the cells were treated with a wide concentration range of asciminib and cell growth and survival were monitored for 48 h. No inhibition was observed for e13a3 and e14a3 for asciminib concentrations of <5 µM (Fig. 2A). Only the highest tested concentration (50 µM) resulted in inhibition of cell proliferation. In contrast, e14a2 showed a half-maximal growth inhibition (GC_50_) of asciminib of ~1 nM (Fig. 2a). These results therefore demonstrated a strong asciminib resistance (~10,000-fold) of e13a3 and e14a3, but not e14a2. Importantly, all three BCR::ABL1 isoforms were highly sensitive to dasatinib with an almost identical GC_50_ of ~2 nM (Fig. 2b). Hence, we observed a selective resistance to allosteric inhibition, while no resistance to ATP-competitive TKIs, such as dasatinib, was observed. We next studied if the observed lack of cell proliferation inhibition by asciminib of e13a3 and e14a3 was due to a lack of inhibition of BCR::ABL1 or a different mechanism. Immunoblotting showed high levels of phospho-tyrosine (pY) for BCR::ABL1 and several other proteins for all three cell lines in the absence of asciminib, but the pY signal of these proteins was only decreased in the e14a2 cell line upon asciminib treatment, whereas no inhibition was seen for e13a3 and e14a3 (Fig. 2c). In line with these results, in e13a3 and e14a3 cells even very high concentrations of asciminib (10 µM, several fold above clinically achievable concentrations) were not able to abolish phosphorylation of STAT5A/B (pY694/pY699), which is critical for leukemogenesis by BCR::ABL1, whereas STAT5 activation was completely inhibited by asciminib in e14a2 cells (Fig. 2d).

To better understand the underlying mechanism of resistance, we hypothesized that the lack of exon 2 is unlikely to cause resistance by a direct effect on asciminib binding to the myristoyl pocket. We therefore performed isothermal titration calorimetry (ITC) experiments to measure binding affinities of asciminib to recombinant BCR::ABL1 fragments containing or lacking the SH3 domain (Fig. 2e). We did not observe a decreased asciminib binding affinity in a BCR::ABL1 protein fragment that lacks a functional SH3 domain when compared to a protein that contains the SH3 domain, in line with our hypothesis (Fig. 2e). This supports an entirely allosteric resistance mechanism, i.e. asciminib binding to the myristoyl pocket is unperturbed, but the lack of a functional SH3 domain in e13a3 and e14a3 prevents BCR::ABL1 kinase inhibition (see Fig. 1a).

In conclusion, we showed a high degree of resistance to asciminib of the BCR::ABL1 e13a3 and e14a3 isoforms that lack ABL1 exon 2. This was due to the lack of a functional SH3 domain, which is required for asciminib action. The very high degree of resistance (~10,000-fold) observed for e13a3 and e14a3 cannot be overcome by higher asciminib doses, in contrast to certain point mutations that were detected in asciminib treated patients. Therefore, CML patients in which the e13a3 and e14a3 breakpoints are identified are predicted to be resistant to asciminib treatment. Despite the small proportion of CML patients with e13a3 and e14a3, the strong rise in asciminib use in clinical practice since its approval, together with the expected approval for first line treatment, will result in a significant number of e13a3 and e14a3 patients that are eligible for treatment with asciminib. Hence, our report is very timely and strongly suggests that preference to an ATP competitive BCR::ABL1 inhibitor should be given over asciminib in patients with e13a3 and e14a3. Of note, other ABL1 fusions that are expressed in Ph-like- and T-cell acute lymphoblastic leukaemias were described to respond to ATP-competitive BCR::ABL1 inhibitors in cells lines and in clinical trials [14]. Some of these ABL1 fusions lack exon 2, such as the RCSD1::ABL1 and SFPQ::ABL1 fusion proteins, which both lack also exon 3, and hence an even bigger part of ABL1 regulatory domains. There are also rare cases of the NUP214::ABL1 lacking exon 2, while most cases include exon 2. These ABL1 fusions are also likely to be resistant to asciminib [15].

In summary, we report that BCR::ABL1 isoforms that lack ABL1 exon 2 (e13a3 and e14a3) are resistant to asciminib inhibition. To our knowledge, this is the first described mechanism of primary asciminib resistance and stressed the importance to screen for BCR::ABL1 isoforms before commencement of treatment.

### Supplementary information

Supplementary Information (Materials and Methods, SI Figures 1 and 2)

## Data Availability

All data generated or analyzed during this study are included in this published article and its supplementary information files.
