# Supplementary material for: The e13a3 (b2a3) and e14a3 (b3a3) BCR::ABL1 isoforms are resistant to asciminib
Source: Leukemia. 2024 Jun 15;38(9):2041–5. doi: 10.1038/s41375-024-02314-7 (PMC11347367; doi:10.1038/s41375-024-02314-7)
Supplement: Supplementary file 1 — Supplementary Information (Materials and Methods, SI Figures 1 and 2) [file 41375_2024_2314_MOESM1_ESM.pdf]

Supplemental Information

Asciminib resistance of e13a3 (b2a3) and e14a3 (b3a3) BCR::ABL1 transcript variants

Inga B. Leske [1] and Oliver Hantschel [1]

[1] Institute of Physiological Chemistry, Faculty of Medicine, Philipps University of Marburg, 35032 Marburg, Germany

Correspondence and requests for materials should be addressed to O.H. (email: oliver.hantschel@uni-marburg.de).

Content

Supplementary Materials and Methods 2
Supplementary Figures 6
References 7

Supplementary Materials and Methods

Antibodies, cell lines and drugs

Antibodies against ABL1 (clone 24-21, ref.[1]) and pY (clone 4G10) were prepared in-house and purified on a ProteinG affinity resin. Antibody against STAT5 (A-9, sc-74442) was purchased from Santa Cruz Biotechnology (Dallas, TX, USA), while antibody against phosphoSTAT5A/B (pY694/pY699; #9359) was purchased from Cell Signaling Technology (Danvers, MA, USA). All primary antibodies were used at 1:1000 dilution either in 3% BSA PBS-T solution or 5% milk TBS-T according to manufacturer instructions. Secondary antibodies used in western blots were obtained from LI-COR (Bad Homburg, Germany): anti-mouse IRDye680 (No. 926–68070) and anti-rabbit IRDye680 (No. 925–68071). Ba/F3 cells (murine pro-B cell line) were purchased by DSMZ (Deutsche Sammlung von Mikroorganismen und Zellkulturen, Braunschweig, Germany, Cat# ACC-300) and cultured in RPMI GlutaMAX supplemented with 10% fetal bovine serum (FBS), 100 µg/ml streptomycin and 100 U/ml penicillin (all ThermoFisher Gibco, Darmstadt, Germany) as well as 10 ng/ml mouse IL-3 (Sigma, Darmstadt, Germany). For drug treatments, asciminib and dasatinib were purchased by Selleckchem (Houston, TX, USA).

Plasmids and cloning

The pMSCV-IRES-eGFP was used as a backbone for the retroviral vectors. *BCR::ABL1*$^{p210/e14a2}$ (ref. [2]) was used as a template to generate *BCR::ABL1*$^{p210/e14a3}$ (by deleting ABL1 exon 2) and *BCR::ABL1*$^{p210/e13a3}$ (by deleting BCR exon14 and ABL1 exon 2). To construct these *BCR::ABL1* deletion mutants, *BCR* and *ABL1* sequences were separately amplified with overhangs using the primers reported in table 1.

Table 1: List of primers used for PCR amplifying of BCR and ABL1 sequences.

		BCR::ABL1^{e13a3}	*BCR::ABL1*^{e14a3}
ABL1	fwd	caataaggaaGGTGAAAAGCTCCGGGTC	gcagagttcaGGTGAAAAGCTCCGGGTC
	rev	TTGGAGTCAGGTTGGGC	TTGGAGTCAGGTTGGGC
BCR	fwd	CTTTATCCAGCCCTCACTC	CTTTATCCAGCCCTCACTC
	rev	gcttttcaccTTCCTTATTGATGGTCAGCG	gcttttcaccTGAACTCTGCTTAAATCCAGTG

Amplified sequences were cloned into the pMSCV-IRES-eGFP vector (cut with EcoRI and SrfI (NEB, Ipswich, MA, USA)) using Gibson Assembly according to the manufacturer's instructions (NEB). All DNA constructs were confirmed by DNA sequencing (Microsynth, Goettingen, Germany).

Retroviral transduction

Retroviral transduction of Ba/F3 cells was performed as previously described.[2] Retroviral supernatants were harvested after 48 h and 72 h and Ba/F3 cells infected twice with supernatant. After one week culturing, GFP-positivity was checked by Flow Cytometry and expression of BCR::ABL1 via immunoblot. Assays were performed after FACS sorting and IL-3 withdrawal.

Cell viability assay

Ba/F3 cells were seeded at 5x10^4 cells/100 µL in 96 well-plates and treated with 10-fold dilutions of asciminib (50 µM - 0.5 pM) and dasatinib (10 µM - 0.1 pM). After 48 hours, relative cell viability was determined using CellTiter-Glo (Promega, Madison, WI, USA) according to manufacturer's instructions. The measured luminescence signal

from mock-treated cells (without drugs) were set to 100% viability, wells containing only cell culture medium (no cells) were used as background normalization. The GC_{50} values were calculated using the Prism software (GraphPad 9.5.0).

Immunoblot analysis

(Auto-)phosphorylation of BCR::ABL1 and downstream targets (e.g., STAT5A/B) in Ba/F3 BCR::ABL1 cell lines was assessed upon treatment with either asciminib (0.01 - 10 µM) or mock (DMSO) for 4 hours at 37 °C. Total protein extraction was done in lysis buffer (50 mM Tris pH 7.5, 150 mM NaCl, 5 mM EDTA, 5 mM EGTA, 1% NP-40) supplemented with 50 mM NaF, 1 mM Vanadate, 1 mM PMSF, 10 µg ml^{-1} TPCK and protease and phosphatase inhibitors (Roche, Basel, Switzerland). Protein concentration was measured using a Bradford assay (Bio-Rad, Hercules, CA, USA; No. 5000006) and equal amounts of proteins were loaded on an SDS-polyacrylamide electrophoresis (PAGE) gel. Transfer to nitrocellulose membranes was performed using a semi-dry blotting system (OWL Peqlab, Erlangen, Germany). Membranes were next incubated overnight at 4 °C with primary antibodies followed by 1 h room temperature incubation with secondary antibodies. Fluorescent detection was performed using the LI-COR Imaging system. Protein expression levels were quantified using the ImageStudio software. To compare phosphorylation levels their relative amounts with respect to BCR::ABL1/STAT5 expression level were calculated.

Protein expression and purification

cDNAs encoding for the human ABL1 SH3-SH2-kinase domain unit (SH3-SH2-KD; residues 63-512) and the human ABL1 SH2-kinase domain unit (SH2-KD; residues 132-515; numbering for the spliceform 1a of the human ABL1 gene is used) was cloned into a pET-series *E. coli* expression vector containing an N-terminal hexa-histidine

affinity tag. ABL1 proteins were co-expressed with the YopH phosphatase in *E.coli* BL21 or ArcticExpress (DE3) (Agilent, Santa Clara, CA, USA) and purified, as previously described.[3] Shortly, cells were harvested by centrifugation, resuspended in 50 mM Tris-HCl pH 7.5/500 mM NaCl/20mM imidazole/5% Glycerol and lysed by three cycles of homogenization at 15,000 psi in Avestin EmulsiFlex-C3 (Mannheim, Germany). Protein purification was carried out by nickel affinity chromatography (His-Trap column; Cytiva, Marlborough, MA, USA) and protein was further purified by anion exchange chromatography on a MonoQ 5/50 GL (Cytiva). Final samples were dialyzed against 50 mM Tris-HCl pH 7.5/500 mM NaCl/5% Glycerol. Protein identity was confirmed by mass spectrometry.

Isothermal Titration Calorimetry (ITC)

ITC measurements were performed on a MicroCal PEAQ-ITC (Malvern Panalytical, Malvern, UK) instrument. Proteins were extensively dialyzed against 50 mM Tris-HCl pH 7.5, 500 mM NaCl, 5% Glycerol and concentration was determined by measuring UV absorbance at 280 nm. Asciminib was dissolved in dimethyl sulfoxide (DMSO, Sigma) to obtain a 10 mM stock solution and diluted to the working concentration of 100 µM in dialysis buffer directly prior to measurements. An equal amount of DMSO was added to the protein solutions (final concentration: 10 µM). Asciminib in the syringe was titrated in 19 steps with 0.4 µL for the first and 2 µL for the other steps to the ABL1 proteins in the calorimetry cell. A titrant-to-buffer control was performed for each measurement and the values subtracted from the experimental data in order to correct for the heat of dilution. Concentrations were adjusted based on assumed affinities and signal strength of the interaction. The MicroCal software was used to determine thermodynamic parameters.

Supplementary Figures

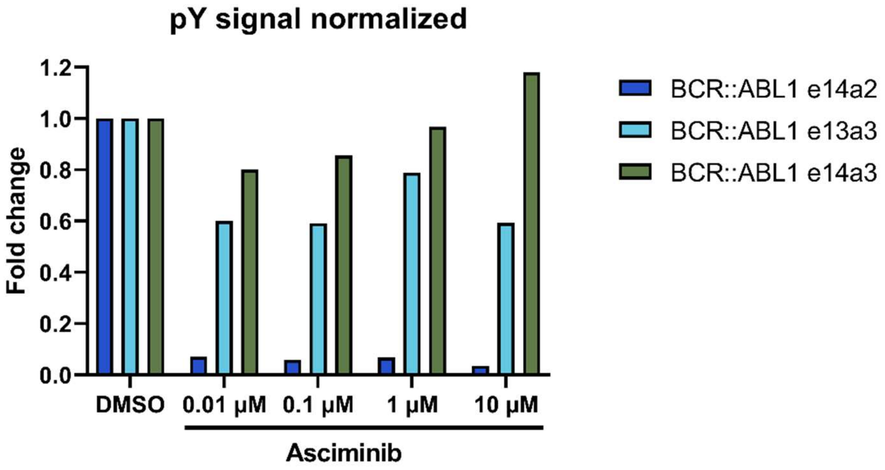

Figure S1: Quantification of immunoblot analysis of tyrosine phosphorylation levels of BCR::ABL1, normalized to BCR::ABL1 expression level, for each cell line after treatment with the indicated asciminib concentrations. Corresponding immunoblots in figure 1c.

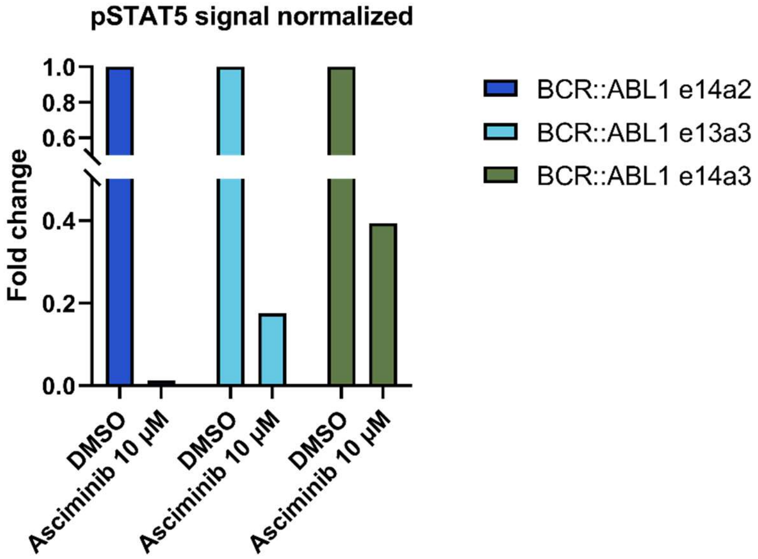

Figure S2: Quantification of immunoblot analysis of STAT5A/B phosphorylation levels, normalized to STAT5A/B expression level, for each cell line after treatment with the indicated asciminib concentration. Corresponding immunoblots in figure 1d.

References

1. Schiff-Maker L, Burns MC, Konopka JB, Clark S, Witte ON, Rosenberg N. Monoclonal antibodies specific for v-abl- and c-abl-encoded molecules. J Virol. 1986;57(3):1182-6.

2. Reckel S, Hamelin R, Georgeon S, Armand F, Jolliet Q, Chiappe D, et al. Differential signaling networks of Bcr-Abl p210 and p190 kinases in leukemia cells defined by functional proteomics. Leukemia. 2017;31(7):1502-12.

3. Seeliger MA, Young M, Henderson MN, Pellicena P, King DS, Falick AM, et al. High yield bacterial expression of active c-Abl and c-Src tyrosine kinases. Protein Sci. 2005;14(12):3135-9.
